# Case of Palpitation of Heart—Hypertrophy of Left Ventricle—Enlargement of Kidneys—Diabetes—Death from Anemia

**Published:** 1841-03-20

**Authors:** 


*Case of palpitation of heart—hypertrophy of left ventricle—enlargement of kidneys—diabetes—death from anemia.* Communicated by Dr. Ashmead.

David R. Corson, of New Hope, æt. 24; height six feet two inches; grew very rapidly but enjoyed excellent health, with the exception of occasional attacks of intermittent fever, and slight erysipelas of the face, to which he was subject for several years, and which continued till his death. In 1834 he suffered considerably from palpitation of the heart; and in 1835 commenced the study of medicine, which confined him very closely, and produced great debility. In February, 1836, his weakness induced him to try active exercise; the effort was followed by hæmoptysis, for which he was freely bled by Prof. Wood, and in ten days was able to return to New Hope in tolerable health, but greatly reduced. He resumed his studies, rode out occasionally, but preserved a pale and cachectic aspect till the summer of 1837. At this period the extraction of a tooth was followed by profuse hæmorrhage, which continued for eight or ten days, and induced such extreme prostration that he was not able to raise his head from the pillow; twenty pounds of blood were supposed to have been lost in two weeks. From this shock he appeared never fully to recover, although his appetite and strength returned in a measure. In the month of September, of the same year, he removed a portion of his uvula, which was elongated, and the operation was followed by great hæmorrhage; in the fall of 1837 he again attended the lectures of the University of Pennsylvania, and graduated with much credit in the spring of 1838.

Upon his return home very much enfeebled, he resumed exercise on horseback; his pulse became very frequent, and he occasionally expectorated a little blood, which appeared to exude from the fauces, and was unattended by cough or pain in the chest, but was connected with frequent ecchymoses of the white of the eyes.

During the winter and spring of 1838–'9, he occasionally attended to practice, but spent the summer of the same year on a farm, using gentle exercise every day, yet his general debility and paleness gradually increased, although his appetite was good, and he himself free from pain, cheerful, and offering no other evidence of disease.

During the spring of 1840 he suffered from an attack of intermittent, which lasted for eight or ten days, and was followed by a relapse, accompanied by violent pain in the bowels, great prostration, with a dark red inflammation of the palate, and oozing of blood from the gums. During his convalescence he exercised his sight very intensely for three days in fine carving, and was suddenly attacked during the night with pain in the head and loss of vision; in a few weeks he was slowly convalescent, and started for Schooley's Mountain, where he remained more than two months with gradual improvement. On his return in the fall, after an attack of diabetes, he frequently rode twenty miles a day; his vision improved so far that he could read ordinary print, but his diabetes increased; the urine was neither saccharine nor albuminous; for this affection he used elix. vitriol, cort. cinchonæ, carb. ferri, and exclusive animal diet.

The amount of urine gradually reached six quarts in twenty-four hours; it was of a pale straw colour, limpid, free from peculiar odour, and not coagulable by heat or nitric acid. The appetite remained unimpaired till within a few days of his death; but the thirst increased, until at last the craving for cold water became insatiable; the blood was so impoverished, that after cutting his finger, his white handkerchief was scarcely stained.

During the last year of his life, every pulsation of the aorta and of the arteries of the extremities was so distinctly felt by the patient, that he imagined the existence of an aneurism in the abdomen.

Although sensible of his situation, his cheerfulness did not desert him, and his intellect remained unimpaired to the end; he was only confined to his room for the last two weeks.

During several months his pulse was 96 while lying down, and rose while erect; on the day before his death it was 104; the weakness gradually increased till it became extreme, and conveyed to him the sensation of over-exhaustion from violent running; it was accompanied by slight spasms on one side of the body, and just before his death by moderate pain in the back, loss of appetite, and occasional vomiting; yet the breathing was never hurried, and he died January 29th, 1841, apparently from extreme debility.

*Autopsy*, *Jan.* 31*st*, *sixty hours after death.*—Weather cold; no discoloration of skin from gravitation of blood; no appearance of decomposition; limbs rigid; surface of an alabaster whiteness and transparency; slight œdema of hands; small ecchymosed spots on chest and shoulder.

*Subcutaneous fat* ¼ inch thick on thorax and abdomen.

*Muscles* of chest and abdomen quite florid and firm.

*Peritoneum* contains half a pint of deep yellow, cloudy serum; external appearance of abdominal viscera natural.

*Stomach* largely distended with three to four pints of fluid and a little gas; the whole mucous membrane is studded over with patches about ¼ inch in diameter, of a dark brown colour, except on the anterior surface, which was not in contact with the fluid contents; in this portion similar patches exist, but offer a bright red colour; they all appear more like ecchymosed spots than the result of an injection of the vessels, but exist in, and not under the mucous membrane. This latter is rather thinner and less consistent than natural.

The *mesenteric glands* very numerous and enlarged, of a dark gray colour, with a tinge of red, offer to the eye and touch a flaccid appearance, giving the idea of a previous greater enlargement and subsequent shrinking; they contained no pus.

*Small intestine.* Mucous membrane of natural colour and consistence. The glands of Brunner are numerous—some of them the size of a pin's head, pale and firm. The glands of Peyer are thickened, of a grayish colour, darker than the mucous membrane.

The duodenum, and six or seven feet of jejunum presented externally a light bluish gray colour; this continued after the contents, of a similar colour, were removed, and the mucus washed off; on close inspection it was clearly seen by the naked eye, to be produced by innumerable, very minute, distinct, dark blue points, which were uniformly strewed over the surface; these appeared to be in the substance of the mucous membrane, and were not removed by slight scraping.

When these points are carefully removed with a knife or the nail, the mucous membrane is perfectly white, smooth, and healthy. Were they in the mouths of the villi? Were they particles of carb. ferri or iod. ferri, exhibited about two weeks before death?

*Large intestine.* Glands of Brunner numerous; vermiform appendix much thickened, of a dark colour, and slightly ulcerated on the under surface; descending colon and rectum covered with numerous small ecchymosed spots.

*Liver* natural size; colon reddish; texture rather soft, not fatty; red and yellow portions very distinct, and in natural proportion; the red considerably injected; very little blood in the large vessels; the biliary ducts contain a bright yellow bile; there spots are found in the centre of the liver, the size of marbles, of a grass green colour; elsewhere natural.

*Gall bladder.* Moderate size, filled with a ropy bile, of a dark brown colour, which stains a white basin of a bright golden yellow.

*Spleen* very small, three by four by one inch, very firm, and almost bloodless.

*Kidneys* surrounded by much fat, large; the left is the largest, five and a half by two and a half by two; opaque white thickened patches are found in the fibrous coat, which separates with great facility; beneath these are seen large ecchymosed patches on the surface of the kidney; renal arteries size of crow-quills; veins four or five times this size—their diameter in a flattened state is half an inch. Pelvis and ureters not dilated or enlarged; lining membrane firm, but ecchymosed. The whole substance of the kidney is firm on pressure; urine accompanied by a thick white (puruloid?) secretion exuded from the papillæ, but remained perfectly distinct from each other; the whole substance of the kidney was charged with urine, which streamed out on cutting or tearing its substance; tubular portion quite pale, and very distinct from the cortical; this latter, at first sight, appeared universally injected; the appearance was due to vessels loaded with dark blood in the interstices of which the acini were pale.

*Bladder* natural; contains several small coagula of blood, and a pint of very pale, thin, almost inodorous urine, not coagulable by heat; mucous membrane unaltered.

The *mediastinum* presents large ecchymosed spots.

The *pleuræ* contain two and a half pints clear, light yellow serum, with a tinge of red; no floculi or signs of recent inflammation.

The *lungs* present numerous large ecchymosed spots on their surface; they are loaded with serum, and at their base are moderately congested with blood; extensive emphysema of edges and apex; bronchial tubes slightly injected; no tubercles.

The apex of the left lung is adherent, and presents a shrivelled appearance and undue firmness; on laying it open, about one dozen calcareous deposits are found from the size of a pea down; these are contained in cysts, lined by a white shining membrane, and crumble readily on pressure.

The *pericardium* contains eight ounces of reddish serum; surface smooth, shining, of a light rose-colour, ecchymosed, but containing no lymph.

*Heart* large, covered with large ecchymosed spots, and surrounded by a considerable degree of fat, especially at its base. *Measurement* at base of ventricles, circumference twelve inches, from base of ventricles to apex five inches; no marks of recent or former inflammation on the surface.

Right auricles large; lining membrane slightly thickened; orifice of coronary vein and opening into ventricle larger than usual; right ventricle contains a white coagulum, but no blood; parietes firm, and rather thicker than natural; its cavity has fully twice its natural capacity; tricuspid valve, when cut open, measures six inches along the line of its attachment; semilunar valves and pulmonary artery unaltered.

Left auricle about same size as right; but the osteum venosum is not enlarged unnaturally.

Left ventricle very large; contains neither blood nor coagulum; parietes very firm and dense, not fatty, rather pale; has a thickness of 7-10ths of an inch; columnæ much thickened; mitral valves natural; measure four and a half inches along their attachment; the semilunar valves of aorta are stained with blood; each presents a rent near and parallel to the loose edge; these rents extend nearly to the point of attachment to the aorta.

Aorta healthy; when cut open measures three and 3-10th inches; the large vessels generally contain *very* little blood; the colour and consistence of the little which is found is natural.

*Head* and *spine* not examined.

